# Unveiling Species Diversity Within Early-Diverging Fungi from China VI: Four *Absidia* sp. nov. (*Mucorales*) in Guizhou and Hainan

**DOI:** 10.3390/microorganisms13061315

**Published:** 2025-06-05

**Authors:** Yi-Xin Wang, Zi-Ying Ding, Xin-Yu Ji, Zhe Meng, Xiao-Yong Liu

**Affiliations:** 1 College of Life Sciences, Shandong Normal University, Jinan 250358, China; wyx13953348060@163.com (Y.-X.W.); 15270343451@163.com (Z.-Y.D.); ji15965902393@163.com (X.-Y.J.); 2 Institute of Microbiology, Chinese Academy of Sciences, Beijing 100101, China

**Keywords:** Mucoromycota, soil-borne fungi, new species, taxonomy, molecular phylogeny

## Abstract

*Absidia* is the most species-rich genus within the family Cunninghamellaceae, with its members commonly isolated from diverse substrates, particularly rhizosphere soil. In this study, four novel *Absidia* species, *A. irregularis* sp. nov., *A. multiformis* sp. nov., *A. ovoidospora* sp. nov., and *A. verticilliformis* sp. nov., were discovered from soil samples collected in southern and southwestern China, using integrated morphological and molecular analyses. Phylogenetic analyses based on concatenated ITS, SSU, LSU, *Act*, and *TEF1α* sequence data reconstructed trees that strongly supported the monophyly of each of these four new taxa. Key diagnostic features include *A. irregularis* (closely related to *A. oblongispora*) exhibiting irregular colony morphology, *A. multiformis* (sister to *A. heterospora*) demonstrating polymorphic sporangiospores, *A. ovoidospora* (forming a clade with *A. panacisoli* and *A. abundans*) producing distinctive ovoid sporangiospores, and *A. verticilliformis* (next to *A. edaphica*) displaying verticillately branched sporangiophores. Each novel species is formally described with comprehensive documentation, including morphological descriptions, illustrations, Fungal Names registration identifiers, designated type specimens, etymological explanations, maximum growth temperatures, and taxonomic comparisons. This work constitutes the sixth instalment in a series investigating early-diverging fungal diversity in China aiming to enhance our understanding of the diversity of fungi in tropical and subtropical ecosystems in Asia. In this paper, the known species of *Absidia* are expanded to 71.

## 1. Introduction

The genus *Absidia* Tiegh. (Mucoromycota Doweld, Mucoromycetes Doweld, Mucorales Dumort., Cunninghamellaceae Naumov ex R.K. Benj.) was established in 1878 and typified with *A. reflexa* [1,2]. It is currently the most numerous genus in the family Cunninghamellaceae, with 67 recognized species (https://www.catalogueoflife.org/data/taxon/LSM, accessed on 2 December 2024). Its taxonomic position has been controversial since its establishment. Initially, this genus was classified in Absidiaceae [3]. Then, Benny et al. transferred it to Mucoraceae in 2001 [4]. Finally, Ashton et al. accommodated it in Cunninghamellaceae in 2009 [5]. With the deepening of research, *Absidia* s.l. was divided into *Lichtheimia* Vuill. (thermotolerant, optimum growth temperature 37–45 °C), *Absidia* s.s. (mesophilic, optimum growth temperature 25–34 °C), and *Lentamyces* Kerst. Hoffm. & K. Voigt (parasitic on other mucoralean fungi, optimum growth temperature 14–25 °C) [6,7,8,9].

Strains of *Absidia* are distributed worldwide, ubiquitous in soil, dung, leaf litter, food, air, etc. [10]. In the GlobalFungi database (https://globalfungi.com/, accessed on 22 December 2024), *Absidia* members are recorded from soil (37,828 records, 44.52% of all records), root (15,865, 18.67%), shoot (11,740, 13.82%), topsoil (6923, 8.15%), rhizosphere soil (2530, 2.98%), deadwood (2253, 2.65%), air (2080, 2.45%), water (1549, 1.82%), litter (1406, 1.65%), mosses (265, 0.41%), lichen (217, 0.25%), coral (72, 0.08%), dust (53, 0.06%), fungal sporocarp (31, 0.04%), and glacial ice debris (3, 0%). In summary, soil and rhizosphere substrates account for approximately 74.32%.

*Absidia coerulea*, the most common species in this genus, plays an important role in bioengineering. It possesses the capability of transforming spirulina biomass into (-)-α-bisabolol [11]. It is able to catalyse the specific C-3 dehydrogenation for derivatives of ginsenoside-Rg_1_, as well as hydroxylation at the 7β and 15α positions, and some metabolites in this species exhibit moderate reversal activity towards multidrug-resistant tumour cells [12].

In order to enhance our understanding of the diversity of fungi in tropical and subtropical soil ecosystems in Asia, eight fungal strains were isolated from soil in the Hainan and Guizhou provinces, the south and southwest regions of China. According to ITS-SSU-LSU-*Act*-*TEF1α* molecular phylogenetic analyses and morphological comparisons, these strains were classified into four new species of *Absidia* and described herein as *A. irregularis* sp. nov., *A. multiformis* sp. nov., *A. ovoidospora* sp. nov., and *A. verticilliformis* sp. nov. This is the sixth report of a serial work on the diversity of Chinese early-diverging fungi [13,14,15,16,17].

## 2. Materials and Methods

### 2.1. Isolation and Morphology

Soil samples were collected in Hainan province in April 2023 and Guizhou province in August 2023, following the methods by Li et al. and Zou et al. [18,19]. Soil sample collection started with removal of surface contaminants using a sterilized stainless-steel shovel. About 100 g of homogenized soil was put into sample bags and labelled with collection date, administrative location, GPS coordinates, and altitude. Strains were obtained from the soil samples by serial dilution spread plate and single spore isolation.

About 1 g of soil samples was mixed with 10 mL of sterile water to prepare 10^−1^ soil suspension. One millilitre of the 10^−1^ suspension was transferred to 9 mL of sterile water to obtain a 10^−2^ soil suspension. In the same way, 10^−3^ and 10^−4^ soil suspensions were made. Approximately 0.2 mL of the final 10^−4^ soil suspension was dispersed evenly with sterilized coating rods on Rose-Bengal Chloramphenicol agar (RBC: peptone 5.00 g/L, glucose 10.00 g/L, KH_2_PO_4_ 1.00 g/L, MgSO_4_·7H_2_O 0.50 g/L, rose red 0.05 g/L, chloramphenicol 0.10 g/L, agar 15.00 g/L) [20] and then cultured in the dark at 25 °C. Once visible, colonies were transferred and further cultured on Potato Dextrose Agar (PDA: glucose 20.00 g/L, potato 200.00 g/L, agar 20.00 g/L, pH 7) [14,21].

Appropriately, 0.1 g of soil samples was evenly sprinkled on the RBC medium with 0.06 mg/mL streptomycin and then incubated in darkness at 25 °C for 2–5 d. When sporangia formed, a sterilized inoculation needle was adopted to pick up a sporangium onto the PDA medium supplemented with 0.06 mg/mL streptomycin.

The maximum growth temperature was determined by a gradient method [13]. The strain was initially cultured at 25 °C for 2 d and then increased by 1 °C per day until there was no growth.

Pure cultivation was applied for observing anamorphs, and pairing experiments were carried out for observing zygospores by adding 0.1% lecithin to PDA and sealing Petri dishes to retain moisture. The morphological characteristics were observed with an optical microscope (Olympus BX53) and photographed with a high-definition colour digital camera (Olympus DP80). Each morphological character was statistically calculated against 30 measurements [22]. All strains were stored at 4 °C with 20% sterilized glycerine. Cultures were deposited in the China General Microbiological Culture Collection Center, Beijing, China (CGMCC) and Shandong Normal University, Jinan, Shandong, China (XG). Strains were deposited in the Herbarium Mycologicum Academiae Sinicae, Beijing, China (Fungarium, HMAS). Taxonomic information for the new taxa was registered in the Fungal Names repository (https://nmdc.cn/fungalnames/, accessed on 14 April 2025).

### 2.2. DNA Extraction and Amplification

Genomic DNA was extracted from mycelia using the CTAB method and GOMag^TM^ Rapid Plant DNA Kit [21]. Information on the primers for PCR amplification is listed in Table 1. Amplification was performed in a final volume of 20 μL, containing 10 μL 2× Hieff Canace^®^ Plus PCR Master Mix (Cat No.10154ES03; Yeasen Biotechnology, Shanghai, China), 0.5 μL of forward and reverse primers each (10 μM; TsingKe, Beijing, China), 1 μL of template genomic DNA (1 μM), and 8 μL of distilled deionised water. Molecular loci, PCR primers, and programmes used in this study are listed in Table 1. PCR products were electrophoresed with 1% agarose gel. DNA fragments were stained with TS-GelRed Nucleic Acid Gel Stain (10,000× in water; TSJ002; Beijing Tsingke Biotech Co., Ltd., Beijing, China) and observed under ultraviolet light. Then, a gel extraction kit (Cat# AE0101-C; Shandong Sparkiade Biotechnology Co., Ltd., Jinan, China) was used for gel recovery. Sanger sequencing was carried out by Biosune Co., Ltd. (Shanghai, China). Consensus sequences were assembled using MEGA v.7.0 [23]. Target sequences in some strains could not be obtained by conventional methods due to heterogeneous gene duplications; thus, they were extracted from genomic data [24]. The genomes were sequenced by Singke Biotech Co., Ltd. (Jinan, China). Genome sequencing was performed using Illumina, and the genomes were assembled using the St. Petersburg genome assembler (SPAdes). All sequences generated in this study were deposited at GenBank under the accession numbers in Table 2. The genome accession numbers of *Absidia ovoidospora* CGMCC 3.27811 and *A. verticilliformis* CGMCC 3.27810 are JBOEPW000000000 and JBOEPV000000000.

Relative sequences were obtained by BLAST search against the NCBI GenBank nucleotide database (https://blast.ncbi.nlm.nih.gov/, accessed on 13 October 2024). SSU, ITS, LSU, *Act*, and *TEF1α* sequences both generated herein and retrieved from GenBank (Table 2) were individually aligned using the MAFFT 7 online service. The aligned matrices were manually proofread and then jointly analysed. The optimal evolutionary model was determined for each partition and included in the analysis using MrModelTest v.2.3 [29]. Phylogenetic history was reconstructed using the maximum likelihood (ML) algorithm with RaxML-HPC2 on XSEDE (8.2.12) and the Bayesian inference (BI) algorithm with MrBayes [30,31,32,33]. Maximum likelihood analysis was performed using the best model with 1000 bootstrap replications. The BI analysis consisted of five million generations with four parallel runs under stopping rules and a sampling frequency of 100 generations. The burn-in score was set to 0.25, and the posterior probability (PP) was determined from the remaining trees. Initial adjustments to the phylogenetic tree were made using FigTree v.1.4.4 (http://tree.bio.ed.ac.uk/software/figtree/, accessed on 23 October 2024), and the finalization was performed using Adobe Illustrator CC 2019 (https://adobe.com/products/illustrator, accessed on 23 October 2024).

## 3. Results

### 3.1. Phylogenetic Analyses

**Figure 1:**
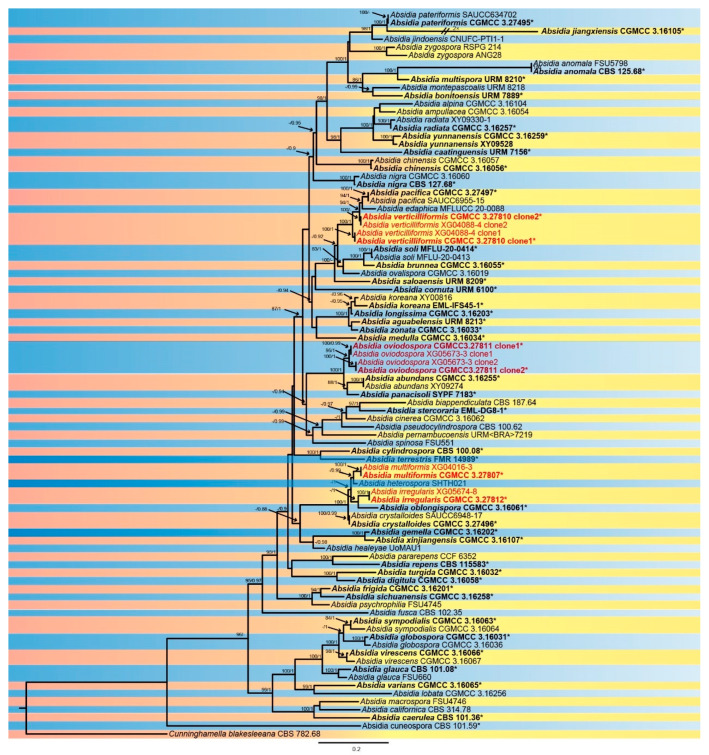
The Bayesian phylogenetic tree of *Absidia* based on ITS-SSU-LSU-Act-TEF1α sequences, with Cunninghamella blakesleeana CBS 782.68 as outgroup. The maximum likelihood bootstrap value (MLBV) ≥ 75 and the Bayesian inference posterior probability (BIPP) ≥ 0.85 are shown at the first and second positions and separated by a slash “/” on relevant nodes. The ex-types or ex-holotypes are in bold and marked with an asterisk “*”, and strains involved in this study are in red. Branches shortened to fit the page are represented by double slashes “//” and folds “×”. The scale at the bottom centre indicates 0.2 substitutions per site.

The sequence matrix included 87 strains in 64 species of *Absidia*, with Cunninghamella blakesleeana CBS 782.68 as an outgroup. A total of 4656 characters comprised ITS rDNA (1–975), SSU rDNA (976–2017), LSU rDNA (2018–3030), *Act* (3031–3689), and *TEF1α* (3690–4656). As many as 2737 characters were constant, while 706 and 1213 among the variable characters were parsimony-uninformative and informative, respectively (Supplementary File S1). MrModelTest suggested that the Dirichlet fundamental frequency and GTR-I-G evolution pattern for all partitions were adopted in Bayesian inference. The topology of the Bayesian tree, consistent with that of the ML tree, was used as a representative to summarise the evolutionary history within the genus *Absidia* (Figure 1), exhibiting the phylogenetic placement of the four new species. A. irregularis is related to A. oblongispora with full supports (MLBV = 100, BIPP = 1.00), and A. ovoidrospora is closely related to A. panacisoli and A. abundans with full supports (MLBV = 100, BIPP = 1.00). A. multiformis is most closely related to A. heterospora with high supports (BIPP = 0.99), and A. verticilliformis is closely related to A. edaphica with robust supports (MLBV = 94, BIPP = 1).

### 3.2. Taxonomy

**Figure 2:**
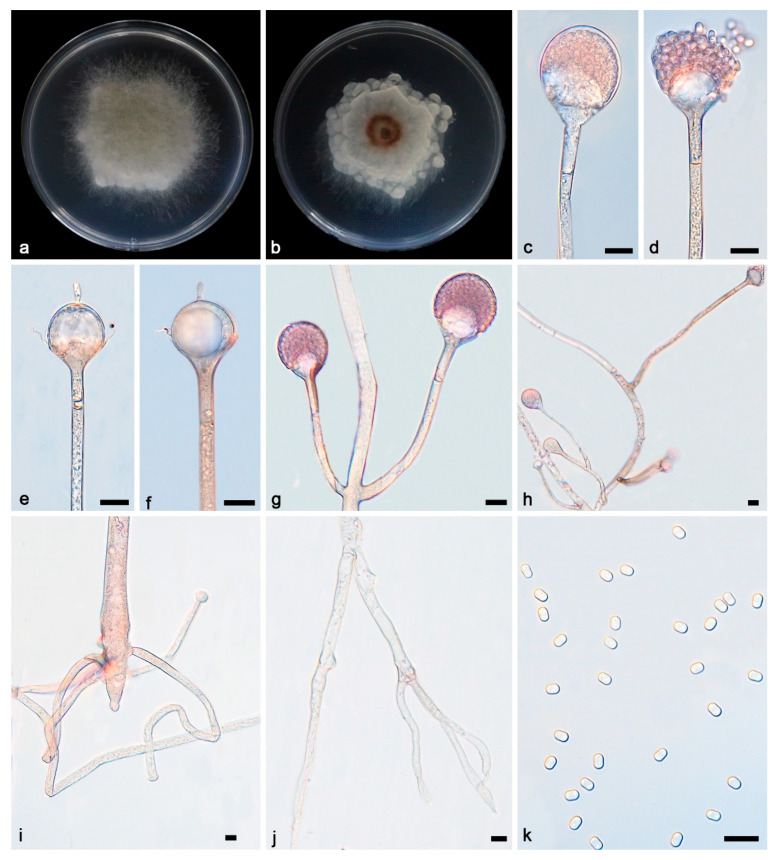
*Absidia irregularis* ex-holotype CGMCC 3.27812. (**a**,**b**) Colonies on PDA ((**a**) obverse; (**b**) reverse); (**c**,**d**) an unbranched sporangiophore with a sporangium; (**e**,**f**) columellae, collars, sporangiospores, and apophyses; (**g**) branched sporangiophores with sporangia; (**h**) branched sporangiophores with columellae, collars, sporangia, and apophyses; (**i**,**j**) rhizoids; (**k**) sporangiospores; bars: (**c**–**k**) 10 μm.

***Absidia irregularis* Yi Xin Wang & X.Y. Liu, sp. nov. Figure 2.**

Fungal Names—FN 572283.

Etymology—The epithet “*irregularis*” (Latin) pertains to irregular colonies.

Type—China, Hainan, Changjiang Li Autonomous County, Bawangling National Forest Park, 19.0859333° N, 109.122752° E, altitude 745.3 m, from soil sample, 9 August 2023, Yi-Xin Wang (Holotype HMAS 353186, ex-holotype strain CGMCC 3.27812 = XG05674-6).

Description—Colonies grow moderately on PDA in darkness at 25 °C for 7 d, reaching 43.2–54.8 mm in diameter, initially white, soon becoming grey to brown, irregular and scaly at the edge, cottony, and reversely white to grey. Hyphae hyaline at first, brownish when mature, branched, irregular, and 10.2–14.5 μm in diameter. Stolons are hyaline, branched, and smooth. Rhizoids are hyaline, branched, irregular, or root-like. Sporangiophores are located on aerial mycelia, hyaline, erect, or slightly curved, unbranched or slightly branched, swollen usually below sporangia, umbellately or sympodially branched, often with a septum below apophyses, 26.5–148.1 × 3.4–5.3 μm. Sporangia oval to subglobose, 27.2–32.4 × 26.5–28.9 μm, hyaline at first and then brown, deliquescent-walled, leaving a collar after releasing sporangiospores. Apophyses are hyaline, smooth, bowl-shaped, 2.9–9.6 × 8.6–19.0 μm. Collars are distinct. Columellae are hyaline or brown, hemispherical, 10.0–19.8 × 9.8–21.0 μm, and protruding (3.5–6.3 × 1.4–2.9 μm) at the top. Protrusions are always slightly contracted in the middle. Sporangiospores are hyaline, smooth, not uniform, mostly cylindrical, 3.6–4.6 × 2.3–2.9 μm. Chlamydospores are present. Zygospores were not observed.

Additional strain examined—China, Hainan, Changjiang Li Autonomous County, Bawangling National Forest Park, 19.0859333° N, 109.122752° E, altitude 745.3 m, from soil sample, 9 August 2023, Yi-Xin Wang (living culture XG05674-8).

GenBank accession numbers—CGMCC 3.27812 (ITS, PQ306325; LSU, PQ289020; *Act*, PQ807209; SSU, PQ799254; *TEF1α*, PV126019), XG05674-7 (ITS, PQ306326; LSU, PQ289021; *Act*, PQ807210; SSU, PQ799255; *TEF1α*, PV126020).

Maximum growth temperature—CGMCC 3.27812 (32 °C), XG05674-7 (32 °C).

Notes—Based on phylogenetic analyses of ITS-SSU-LSU-*Act*-*TEF1α* sequences, the two isolates of the new species *Absidia irregularis* formed an independent clade with full supports (MLBV = 100, BIPP = 1; Figure 1), which is closely related to *A. oblongispora* (BIPP = 1; Figure 1). This new species differs morphologically from *A. oblongispora* in apophysis and columella [34]. The new species is bigger than *A. oblongispora* in apophysis, 2.9–9.6 × 8.6–19.0 μm vs. 3.5–6.5 × 3.5–7.5 μm. The new species is also bigger than *A. oblongispora* in columellae, 10.0–19.8 × 9.8–21.0 μm vs. 7.0–15.0 × 8.5–16.5 μm. Combining the morphological and molecular phylogenetic analyses, the two isolates were classified as a new taxon, allied to *A. oblongispora*.

**Figure 3:**
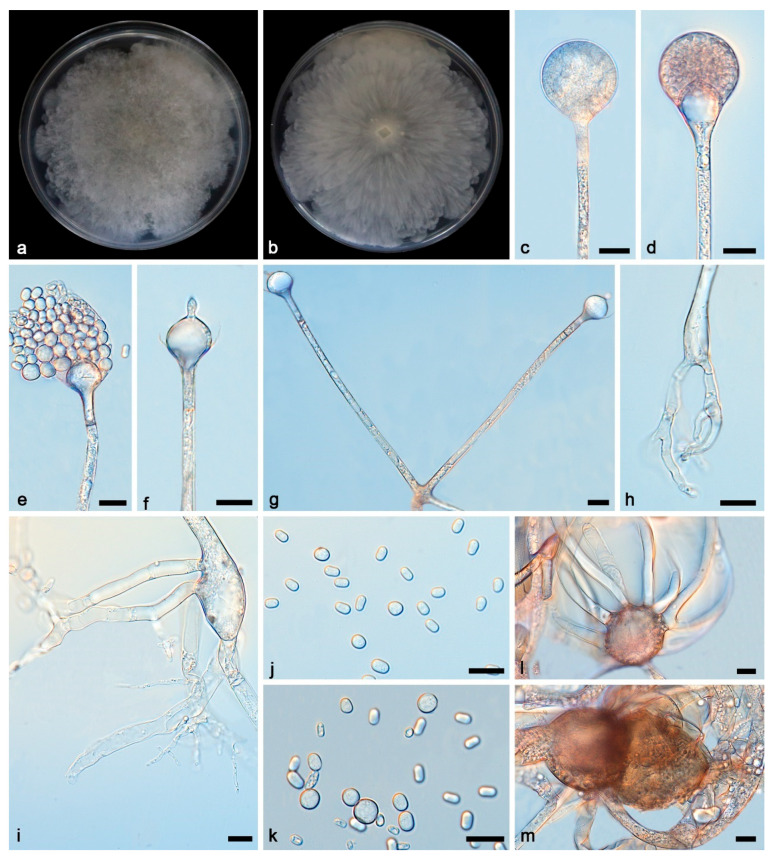
*Absidia multiformis* ex-holotype CGMCC 3.27807. (**a**,**b**) Colonies on PDA ((**a**) obverse; (**b**) reverse); (**c**,**d**) an unbranched sporangiophore with a sporangium; (**e**,**f**) columellae, collars, sporangiospores, and apophyses; (**g**) branched sporangiophores with columellae, collars, sporangium, and apophyses; (**h**,**i**) rhizoids; (**j**,**k**) sporangiospores; (**l**,**m**) zygospores; bars: (**c**–**m**) 10 μm.

***Absidia multiformis* Yi Xin Wang & X.Y. Liu, sp. nov. Figure 3.**

Fungal Names—FN 572285.

Etymology—The epithet “*multiformis*” (Latin) pertains to polymorphic sporangiospores.

Type—China, Hainan, Lingshui Li Autonomous County, 18.6958768° N, 109.9407998° E, altitude 151.6 m, from soil sample, 23 April 2023, Yi-Xin Wang (Holotype HMAS 353194, ex-holotype strain CGMCC 3.27807 = XG04016-2).

Description—Colonies grow moderately on PDA in darkness at 25 °C for 7 d, reaching 82.2–90.0 mm in diameter, initially white, soon becoming grey to brown, irregular at the edge, cottony, and reversely white to grey. Hyphae are hyaline at first, brownish when mature, branched, irregular, and 4.2–12.3 μm in diameter. Stolons are hyaline, branched, and smooth. Rhizoids are hyaline, branched, irregular, or root-like. Sporangiophores on aerial mycelia are hyaline, erect, or slightly curved, unbranched, or slightly branched, swollen usually below sporangia, often with a septum below apophyses, 49.9–125.4 × 2.9–3.7 μm. Sporangia are oval to subglobose, and 20.1–36.1 × 23.1–36.3 μm, hyaline at first and then brown, and deliquescent-walled, leaving a collar after releasing sporangiospores. Apophyses are hyaline, smooth, shallow mouth bowl-shaped, and 2.5–7.9 × 8.0–19.8 μm. Collars are distinct. Columellae are hyaline or brown, hemispherical, 5.3–19.4 × 8.3–22.9 μm, with long or short cylindrical protrusions at top, and 1.7–6.4 × 1.1–4.0 μm. Sporangiospores are hyaline, smooth, not uniform, mainly cylindrical, and 4.5–6.1 × 2.5–4.4 μm, and some are ovoid, 4.4–5.8 × 3.9–6.1 μm, occasionally subglobose, and 4.9–7.2 μm. Chlamydospores are present. Zygospores are present, not uniform.

Additional strain examined—China, Hainan, Lingshui Li Autonomous County, 18.6958768° N, 109.9407998° E, altitude 151.6 m, from soil sample, 23 April 2023, Yi-Xin Wang (living culture XG04016-3).

GenBank accession numbers—CGMCC 3.27807 (ITS, PQ306319; LSU, PQ803168; *Act*, PQ807203; SSU, PQ799260; *TEF1α*, PV126021), XG0 4016-3 (ITS, PQ306320; LSU, PQ803169; *Act*, PQ807204; SSU, PQ799261; *TEF1α*, PV126022).

Maximum growth temperature—CGMCC 3.27807 (32 °C), XG0 4016-3 (32 °C).

Notes—Based on phylogenetic analyses of ITS-SSU-LSU-*Act*-*TEF1α* sequences, the two isolates of the new species *Absidia multiformis* formed an independent clade with full supports (MLBV = 100, BIPP = 1; Figure 1), which is closely related to *A. heterospora* (BIPP = 0.99; Figure 1). This new species differs morphologically from *A. heterospora* in sporangiophore, sporangium, apophysis, and columella [35]. It differs from *A. heterospora* by narrower and shorter sporangiophores, 49.9–125.4 × 2.9–3.7 μm vs. 230–1700 × 4.3–10.0 µm. It is smaller than *A. heterospora* in sporangia, 20.1–36.3 μm vs. 15.0–55.0 µm. In columellae, it differs from *A. heterospora* by more shapes and smaller size. In detail, it is hemispherical and 5.3–19.4 × 8.3–22.9 μm, while *A. heterospora* is regularly dorsiventrally flattened and 10.5–34 µm in diameter. Combining morphological and molecular phylogenetic analyses, the two isolates were classified as a new taxon, allied to *A. heterospora*.

**Figure 4:**
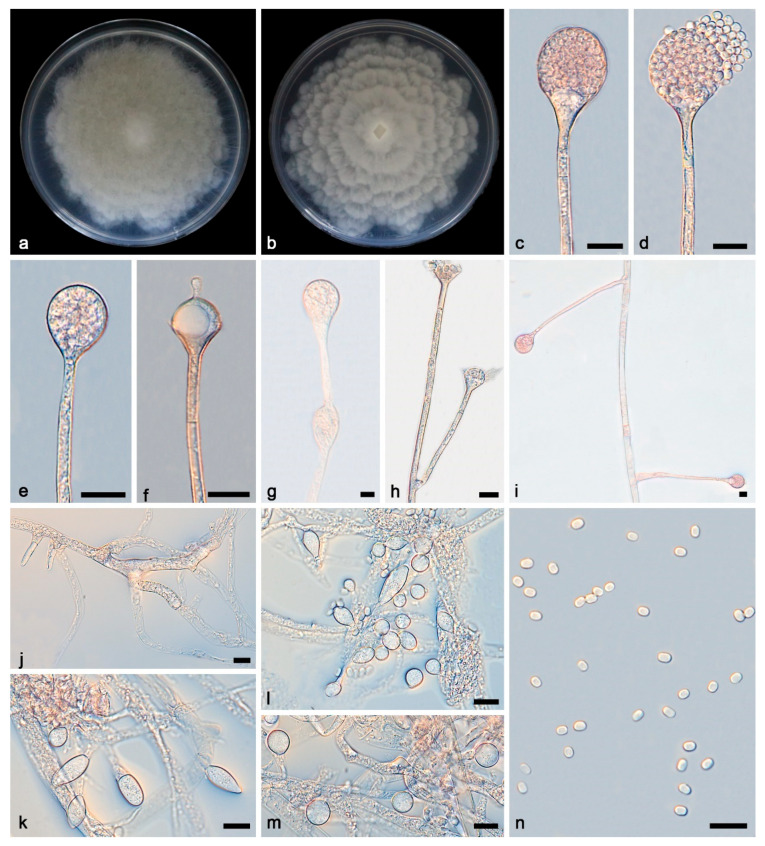
*Absidia ovoidospora* ex-holotype CGMCC 3.27811. (**a**,**b**) Colonies on PDA ((**a**) obverse; (**b**) reverse); (**c**–**e**) an unbranched sporangiophore with a sporangium; (**f**) columellae, collars, sporangiospores, and apophyses; (**g**) unbranched sporangiophores with swelling and sporangium; (**h**) branched sporangiophores with columellae, collars, and apophyses; (**i**) branched sporangiophores with sporangia; (**j**) rhizoids; (**k**–**m**) giant cells; (**n**) sporangiospores; bars: (**c**–**n**) 10 μm.

***Absidia ovoidospora* Yi Xin Wang & X.Y. Liu, sp. nov. Figure 4.**

Fungal Names—FN 572284.

Etymology—The epithet “*ovoidospora*” (Latin) pertains to ovoid sporangiospores.

Type—China, Hainan, Changjiang Li Autonomous County, Bawangling National Forest Park, 25.905722° N, 107.279063° E, altitude 745.3 m, from soil sample, 9 August 2023, Yi-Xin Wang (Holotype HMAS 353185, ex-holotype strain CGMCC 3.27811 = XG05673-2).

Description—Colonies grow fast on PDA in darkness at 25 °C for 7 d, reaching 90 mm in diameter, initially white, soon becoming grey to brown, irregular at the edge, cottony, and reversely white to grey. Hyphae are hyaline at first, brownish when mature, branched, irregular, and 5.8–13.7 μm in diameter. Stolons are hyaline, branched, and smooth. Rhizoids are hyaline, branched, irregular, or root-like. Sporangiophores are on aerial mycelia, hyaline, erect or slightly curved, unbranched or slightly branched, swollen usually below sporangia, umbellately or sympodially branched, often with a septum below apophyses, and 45.3–355.3 × 2.5–4.0 μm. Sporangia are oval to subglobose, 13.6–29.0 × 13.3–28.5 μm, hyaline at first and then brown, deliquescent-walled, leaving a collar after releasing sporangiospores. Apophyses are hyaline, smooth, bowl-shaped and long funnel-shaped, and 4.1–6.9 × 8.6–10.9 μm. Columellae are hyaline or brown, hemispherical with a short or long protruding at the top, and 7.9–12.9 × 9.0–11.6 μm. Protrusions are always slightly contracted in the middle and 1.9–4.6 × 1.7–2.6 μm. Sporangiospores are hyaline, smooth, not uniform, mostly ovoid, and 3.2–3.8 × 2.4–3.1 μm, and some are cylindrical and 3.4–4.6 × 2.2–3.0 μm μm. Chlamydospores are present. Zygospores were not observed.

Additional strains examined—China, Hainan, Changjiang Li Autonomous County, Bawangling National Forest Park, 25.905722° N, 107.279063° E, altitude 745.3 m, from soil sample, 9 August 2023, Yi-Xin Wang (living culture XG05673-3).

Genome accession numbers—JBOEPW000000000.

GenBank accession numbers—CGMCC 3.27811 clone1 (ITS, PQ306327; LSU, PQ803164; *Act*, PQ807207; SSU, PQ799256; *TEF1α*, PV126015), CGMCC 3.27811 clone2 (ITS, PV069753; LSU, PQ803165; *Act*, PV126023; SSU, PQ799257; *TEF1α*, PV126017), XG05673-3 clone 1 (ITS, PQ306328; LSU, PQ803166; *Act*, PQ807208; SSU, PQ799258; *TEF1α*, PV126016), XG05673-3 clone 2 (ITS, PV069754; LSU, PQ803167; *Act*, PV126024; SSU, PQ799259; *TEF1α*, PV126018).

Maximum growth temperature—CGMCC 3.27811 (30 °C), XG05673-3 (30 °C).

Notes—Based on phylogenetic analyses of ITS-SSU-LSU-*Act*-*TEF1α* sequences, the new species *Absidia ovoidospora* formed two sister clades with high supports (MLBV = 96, BIPP = 1; Figure 1), which are closely related to *A. panacisoli* and *A. abundans* with full supports (MLBV = 100, BIPP = 1; Figure 1). These two clades resulted from two clones. This new species differs morphologically from *A. oblongispora* in sporangiophore and sporangium [36]. The new species differs from *A. panacisoli* by a wider sporangiophore, 2.5–4.0 μm vs. 1.7–2.8 μm. In sporangia, the new species is bigger than *A. panacisoli*, 13.6–29.0 × 13.3–28.5 μm vs. 13.0–21.5 × 9.4–15.5 μm. This new species differs morphologically from *A. abundans* in sporangiophore, sporangium, and columella [9]. The new species differs from *A. abundans* by bigger sporangiophores, 45.3–355.3 × 2.5–4.0 μm vs. 35.0–170.0 × 2.0–3.5 μm. The new species is bigger than *A. abundans* in sporangia, 13.6–29.0 × 13.3–28.5 μm vs. 8.0–16.5 × 8.5–16.0 μm. In columellae, the new species is bigger than *A. abundans*, 7.9–12.9 × 9.0–11.6 μm vs. 4.5–10.0 × 3.5–8.0 μm. Combining morphological and molecular phylogenetic analyses, the two were classified isolates as a new taxon, allied with *A. panacisoli* and *A. abundans*.

**Figure 5:**
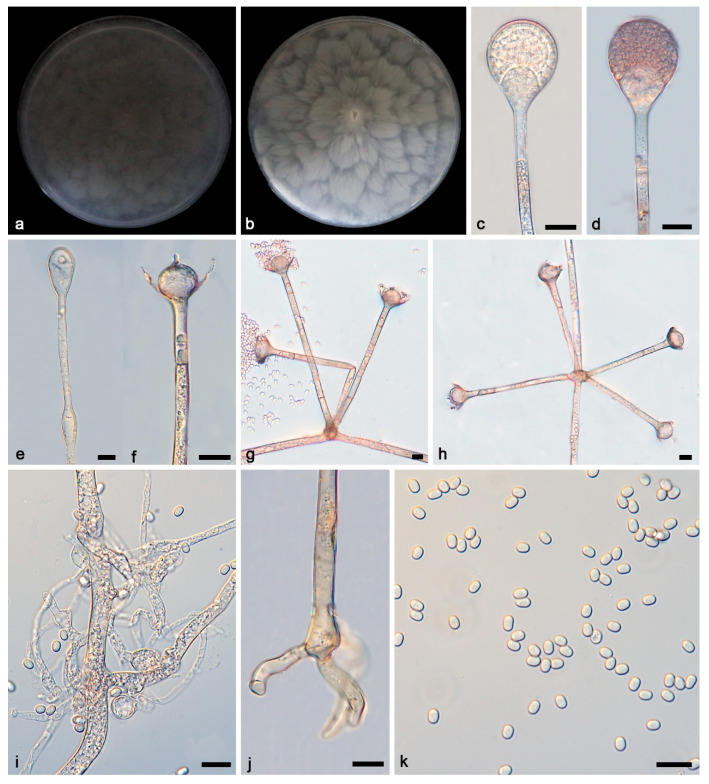
*Absidia verticilliformis* ex-holotype CGMCC 3.27810. (**a**,**b**) Colonies on PDA ((**a**) obverse; (**b**) reverse); (**c**–**e**) an unbranched sporangiophore with a sporangium; (**f**) columellae, collars, sporangiospores, and apophyses; (**g**,**h**) branched sporangiophores with columellae, collars, sporangiospores, and apophyses; (**i**,**j**) rhizoids; (**k**) sporangiospores; bars: (**c**–**k**) 10 μm.

***Absidia verticilliformis*, Yi Xin Wang & X.Y. Liu sp. nov. Figure 5.**

Fungal Names—FN 572287.

Etymology—The epithet “*verticilliformis*” (Latin) pertains to verticillate branches of sporangiophores.

Type—China, Hainan, Sanya City, Jiyang District, G224 Haiyu Middle Line, 18.391817° N, 109.641068° E, altitude 193.0 m, from soil sample, 24 April 2023, Yi-Xin Wang (Holotype HMAS 353183, ex-holotype strain CGMCC 3.27810 = XG04088-3).

Description—Colonies grow fast on PDA in darkness at 25 °C for 7 days, reaching 90 mm in diameter, initially white, soon becoming grey to brown, irregular at the edge, cottony, and reversely white to grey, growing outward in a petal shape. Hyphae are hyaline at first, brownish when mature, branched, and 4.4–8.9 μm in diameter. Stolons are hyaline, branched, and smooth. Rhizoids are hyaline, branched, irregular, or root-like. Sporangiophores are located on aerial mycelia, hyaline, erect or slightly curved, unbranched or slightly branched, and mostly umbellately branched, and swelling is usually present below the sporangia, often with a septum below apophyses, 22.3–368.7 × 3.3–4.6 μm. Sporangia are oval to subglobose, 15.7–29.0 × 17.5–26.0 μm, hyaline at first and then brown, and deliquescent-walled, mostly leaving a collar after releasing sporangiospores. Apophyses are hyaline, smooth, shallow mouth bowl-shaped, and 4.5–14.4 × 11.1–20.4 μm. Collars are distinct. Columellae are hyaline or brown, hemispherical, with tips with short or long cylindrical protrusions, and 12.6–25.6 × 14.7–26.2 μm. Protrusions are 3.1–6.3 × 1.8–2.2 μm. Sporangiospores are hyaline, smooth, not uniform, mostly ovoid, and 3.4–4.3 × 2.3–3.1 μm, and some are cylindrical, 3.9–4.4 × 2.1–3.0 μm. Chlamydospores are present. Zygospores were not observed.

Additional strain examined—China, Hainan, Sanya City, Jiyang District, G224 Haiyu Middle Line, 18.391817° N, 109.641068° E, altitude 193.0 m, from soil sample, 24 April 2023, Yi-Xin Wang (living culture XG04088-4).

Genome accession number—JBOEPV000000000.

GenBank accession numbers—CGMCC 3.27810 clone 1 (ITS, PQ306315; LSU, PQ803170; *Act*, PQ807205; SSU, PQ799262; *TEF1α*, PV126011), CGMCC 3.27810 clone 2 (ITS, PV069755; LSU, PQ803171; *Act*, PV126025; SSU, PQ799263; *TEF1α*, PV126013), XG04088-4 clone1 (ITS, PQ306316; LSU, PQ803172; *Act*, PQ807206; SSU, PQ799264; *TEF1α*, PV126012), XG04088-4 clone2 (ITS, PV069756; LSU, PQ803173; *Act*, PV126026; SSU, PQ799265; *TEF1α*, PV126014).

Maximum growth temperature—CGMCC 3.27810 (34 °C), XG04088-4 (34 °C).

Notes—Based on phylogenetic analyses of ITS-SSU-LSU-*Act*-*TEF1α* sequences, the two isolates of the new species *Absidia verticilliformis* formed two sister clades with full supports (MLBV = 100, BIPP = 1; Figure 1), which is closely related to *A. edaphica* (MLBV = 94, BIPP = 1; Figure 1). This new species differs morphologically from *A. edaphica* in its sporangia, columellae, and sporangiospores [37]. The new species is smaller than *A. edaphica* in sporangia, 15.7–29.0 × 17.5–26.0 μm vs. 30.5–35.5 × 24–27 μm. In columellae, the new species is bigger than *A. edaphica*, 12.6–25.6 × 14.7–26.2 μm vs. 5–9.5 × 6.5–20 μm. The new species is smaller than *A. edaphica* in sporangiospores, 3.4–4.4 × 2.1–3.1 μm vs. 3.5–5.5 × 2–3.5 μm. Combining morphological and molecular phylogenetic analyses, the two isolates were classified as a new taxon, allied to *A. edaphica*.

## 4. Discussion

The genus *Absidia* was established nearly 150 years ago. Between 1878 and 2010, the taxonomic status of the genus was changed several times, and the species of the genus were divided according to the optimum growth temperature, phylogenetic relationships, and microscopic morphology. Finally, *Absidia* s. l. has been divided into three genera, namely *Absidia* s. s., *Lichtheimia*, and *Lentamyces* [7,8]. Nowadays, *Absidia* is the most species-rich genus in the family Cunninghamellaceae. Together with the four new species proposed in this study, the world diversity of *Absidia* s.s. reaches 71 recognized species.

According to Hoffmann’s study [6], *Absidia* s.s. is not thermotolerant, with an optimal growth temperature above 30 °C, and does not grow above 37 °C, rapidly growing. The sporangiophore has a subsporangial septum, and the zygospore has sterile hair-like, mycelial appendages on the suspensors. It does not parasitize on other Mucorales fungi; *Lichtheimia* is thermotolerant, with an optimal temperature above 34 °C and growth above 37 °C, rapidly growing. The sporangiophore has a subsporangial septum, and zygospore has no mycelial appendages on the suspensors. It does not parasitize on other Mucorales fungi; *Lentamyces* is not thermotolerant, and its temperature maximum is below 30 °C, slowly growing, sporangiophores with subsporangial septum, potentially parasitic on other Mucorales, homothallic, and warty zygospores without appendaged suspensors.

Phylogenetically, based on the ITS sequence, a maximum likelihood method (ML) tree was reconstructed for main species of the genus *Absidia* s.s., *Lichtheimia*, and *Lentamyces*, as well as the four new species proposed herein. Three groups with relatively high supports emerged, which was consistent with the division mentioned above, and the four new species in this article were clustered in the *Absidia* s.s. group (Supplementary File S3). Physiologically, thermal tolerance thresholds of fungal strains were determined using a temperature gradient cultivation technique. Growth characterization revealed distinct maximum growth temperatures for the four *Absidia* species: *A. ovoidospora* (30 °C), *A. irregularis* (32 °C), *A. multiformis* (34 °C), and *A. verticilliformis* (34 °C). These thermal parameters align with the established physiological profile of *Absidia* s.s. (maximum growth temperature at 30–37 °C) [7]. Morphologically, the sporangiophore has a subsporangial septum of the four species, and *Absidia multiformis* captured the presence of sterile hair-like, mycelial appendages on the suspensors of zygospore.

Based on the above phylogenetic, morphological, and optimal growth temperature evidence, the four species in this paper belong to *Absidia* s.s.

Prior to 2020, the phylogenetic tree of this genus was predominantly reconstructed utilizing the combination of ITS and LSU sequences [36]. Subsequently, the SSU sequence and the protein sequences *Act* and *TEF1α* were gradually added, yielding results that were largely in line with previous findings [13,17,37,38]. Aiming at the problem of overlapping peaks in the PCR amplification of *Act* protein sequences of some species, genomic sequencing was performed on them in this paper, and the corresponding sequences were extracted from the assembled genomes. It is speculated that the chromatogram overlapping problem is due to the existence of duplicate copies in this section of the gene sequence, and this problem has also been confirmed through genomic extraction sequences. Extracting sequences by assembling genomes might become a solution to the problem of overlapping in PCR sequencing of some species.

In this study, the phylogenetic history was inferred using ITS-SSU-LSU-*Act*-*TEF1α*, and newly isolated strains were grouped into four individual clades with high supports, namely *Absidia irregularis* sp. nov., *A. multiformis* sp. nov., *A. ovoidospora* sp. nov., and *A. verticilliformis* sp. nov.

*Absidia irregularis* is related to *A. oblongispora* with high supports (MLBV = 100, BIPP = 1; Figure 1), while distinguished by bigger apophyses and columellae. *Absidia multiformis* is most closely related to *A. heterospora* with high support (BIPP = 0.99; Figure 1). Morphologically, *A. multiformis* has narrower and shorter sporangiophores, smaller sporangia size, and different columellae shape and size. *Absidia ovoidospora* is closely related to *A. panacisoli* and *A. abundans*. However, compared with *A. panacisoli*, *A. ovoidospora* had wider sporangiophore width and a larger sporangium. At the same time, compared with *A. abundans*, the microscopical measurements of the sporangiophore, sporangium, and columella of *A. ovoidospora* were larger. *A. verticilliformis* is closely related to *A. edaphica*. *A. verticilliformis* differs from *A. edaphica* by smaller sporangia, bigger columellae, and smaller sporangiospores.

In summary, the molecular phylogenetic and morphological results both support the identification of the four new species. These findings further enhance our understanding of mucoralean biodiversity in Asian tropical and subtropical ecosystems.

## Figures and Tables

**Table 1:** Molecular loci, PCR primers, and programmes used in this study.

Loci	PCR Primers	Sequence (5′–3′)	PCR Cycles	Reference
*Act*	ACT-512F	ATG TGC AAG GCC GGT TTC GC	95 °C 3 min; (95 °C 1 min, 55 °C 1 min, 72 °C 1 min) × 30 cycles; 72 °C 10 min	[25]
	ACT-783R	TAC GAG TCC TTC TGG CCC AT		
ITS	ITS5	GGA AGT AAA AGT CGT AAC AAG G	95 °C 5 min; (95 °C 30 s, 55 °C 30 s, 72 °C 1 min) × 35 cycles; 72 °C 10 min	[26]
	ITS4	TCC TCC GCT TAT TGA TAT GC		
LSU	LR0R	GTA CCC GCT GAA CTT AAG C	95 °C 5 min; (95 °C 50 s, 47 °C 30 s, 72 °C 1.5 min) × 35 cycles; 72 °C 10 min	[27]
	LR7	TAC TAC CAC CAA GAT CT		
SSU	NS1	GTA GTC ATA TGC TTG TCT C C	95 °C 5 min; (94 °C 60 s, 54 °C 50 s, 72 °C 60 s) × 37 cycles; 72 °C 10 min	[26]
	NS4	CTT CCG TCA ATT CCT TTA AG		
*TEF1α*	EF1-728F	CAT CGA GAA GTT CGA GAA GG	95 °C 5 min; (95 °C 30 s, 55 °C 60 s, 72 °C 1 min) × 30 cycles; 72 °C 10 min	[28]
	EF2	GGA RGT ACC AGT SAT CAT GTT		

**Table 2:** Information on strains used in this study.

Species	Strains	GenBank Accession Numbers				
		ITS	LSU	*TEF1α*	*Act*	SSU
***Absidia abundans***	CGMCC 3.16255 *	NR_182590	ON074683	n.a.	n.a.	n.a.
*A. abundans*	XY09274	ON074696	ON074682	n.a.	n.a.	n.a.
*A. aguabelensis*	URM 8213 *	NR_189383	NG_241934	n.a.	n.a.	n.a.
*A. alpina*	CGMCC 3.16104	OL678133	n.a.	n.a.	n.a.	n.a.
*A. ampullacea*	CGMCC 3.16054	MZ354138	MZ350132	n.a.	n.a.	n.a.
*A. anomala*	CBS 125.68 *	MH859085	MH870799	n.a.	n.a.	n.a.
*A. anomala*	FSU5798	EF030523	n.a.	n.a.	EF030535	n.a.
*A. biappendiculata*	CBS 187.64	MZ354153	MZ350147	MZ357420	MZ357438	
*A. bonitoensis*	URM 7889 *	MN977786	MN977805	n.a.	n.a.	n.a.
*A. brunnea*	CGMCC 3.16055 *	MZ354139	MZ350133	MZ357403	MZ357421	n.a.
*A. caatinguensis*	URM 7156 *	NR_154704	NG_058582	n.a.	n.a.	n.a.
*A. caerulea*	CBS101.36 *	MH855718	MH867230	n.a.	n.a.	n.a.
*A. californica*	CBS 314.78	JN205816	MH872902	n.a.	n.a.	n.a.
*A. chinensis*	CGMCC 3.16057	MZ354141	MZ350135	n.a.	MZ357422	n.a.
*A. chinensis*	CGMCC 3.16056 *	MZ354140	MZ350134	n.a.	n.a.	n.a.
*A. cinerea*	CGMCC 3.16062	MZ354146	MZ350140	MZ357407	MZ357427	n.a.
*A. cornuta*	URM 6100 *	NR_172976	MN625255	n.a.	n.a.	n.a.
*A. crystalloides*	CGMCC3.27496 *	PP377803	PP373736	PP790574	PP790582	PP779723
*A. crystalloides*	SAUCC693201	PP377804	PP373737	PP790573	PP790581	PP779722
*A. cuneospora*	CBS 101.59 *	MH857828	MH869361	n.a.	n.a.	n.a.
*A. cuneospora*	FSU5890	EF030524	n.a.	n.a.	EF030533	n.a.
*A. cylindrospora*	CBS 100.08	JN205822	JN206588	n.a.	n.a.	n.a.
*A. digitula*	CGMCC 3.16058 *	MZ354142	MZ350136	MZ357404	MZ357423	n.a.
*A. edaphica*	MFLUCC 20-0088	NR_172305	NG_075367	n.a.	MT410739	NG_074951
*A. frigida*	CGMCC 3.16201 *	NR_182565	OM030223	n.a.	n.a.	n.a.
*A. fusca*	CBS 102.35 *	NR_103625	NG_058552	n.a.	n.a.	n.a.
*A. gemella*	CGMCC 3.16202 *	OM108488	OM030224	n.a.	n.a.	n.a.
*A. glauca*	CBS 101.08 *	MH854573	MH866105	n.a.	n.a.	n.a.
*A. glauca*	FSU660	AY944879	EU736302	EU736248	EU736225	EU736275
*A. globospora*	CGMCC 3.16031 *	NR_189829	MW671544	MZ357412	MZ357431	n.a.
*A. globospora*	CGMCC 3.16036	MW671539	MW671546	MZ357414	MZ357433	n.a.
*A. healeyae*	UoMAU1	n.a.	MT436027	n.a.	n.a.	n.a.
*A. heterospora*	SHTH021	JN942683	JN982936	n.a.	n.a.	JQ004928
***A. *** ***irregularis***	**CGMCC 3.27812** *	**PQ306325**	**PQ289020**	**PV126019**	**PQ807209**	**PQ799254**
***A. *** ***irregularis***	**XG05674-7**	**PQ306326**	**PQ289021**	**PV126020**	**PQ807210**	**PQ799255**
*A. jiangxiensis*	CGMCC 3.16105 *	OL678134	PP780377	n.a.	n.a.	n.a.
*A. jindoensis*	CNUFC-PTI1-1	MF926622	MF926616	MF926513	MF926510	MF926626
*A. koreana*	EML-IFS45-1 *	KR030062	KR030056	KR030060	KR030058	KT321298
*A. koreana*	XY00816	OL620083	ON123771	n.a.	n.a.	n.a.
*A. lobata*	CGMCC 3.16256	ON074690	ON074679	n.a.	n.a.	n.a.
*A. longissima*	CGMCC 3.16203 *	NR_182566	OM030225	n.a.	n.a.	n.a.
*A. macrospora*	FSU4746	AY944882	EU736303	EU736249	AY944760	EU736276
*A. medulla*	CGMCC 3.16034	NR_189832	MW671549	MZ357417	MZ357436	n.a.
*A. montepascoalis*	URM 8218	NR_172995	n.a.	n.a.	n.a.	n.a.
***A. *** ***multiformis***	CGMCC 3.27807 *	**PQ306319**	**PQ803168**	**PV126021**	**PQ807203**	**PQ799260**
***A. *** ***multiformis***	**XG04016-3**	**PQ306320**	**PQ803169**	**PV126022**	**PQ807204**	**PQ799261**
*A. multispora*	URM 8210 *	MN953780	MN953782	n.a.	n.a.	n.a.
*A. nigra*	CBS 127.68 *	NR_173068	MZ350146	MZ357419	MZ357437	n.a.
*A. nigra*	CGMCC 3.16060	MZ354144	MZ350138	MZ357406	MZ357425	n.a.
*A. oblongispora*	CGMCC 3.16061	MZ354145	MZ350139	n.a.	MZ357426	n.a.
*A. ovalispora*	CGMCC 3.16019	NR_176748	MW264131	n.a.	n.a.	n.a.
***A. *** ***ovoidospora***	**CGMCC 3.27811 clone1** *	**PQ306327**	**PQ803164**	**PV126015**	**PQ807207**	**PQ799256**
***A. ovoidospora***	**CGMCC 3.27811 clone2** *	**PV069753**	**PQ803165**	**PV126017**	**PV126023**	**PQ799257**
***A. ovoidospora***	**XG05673-3 clone1**	**PQ306328**	**PQ803166**	**PV126016**	**PQ807208**	**PQ799258**
***A. ovoidospora***	**XG05673-3 clone2**	**PV069754**	**PQ803167**	**PV126018**	**PV126024**	**PQ799259**
*A. pacifica*	CGMCC3.27497 *	PP377802	**PP373735**	PP839793	PP790579	PP779720
*A. pacifica*	SAUCC413601	PP377801	PP373734	PP839794	PP790580	PP779721
*A. panacisoli*	SYPF 7183 *	MF522181	MF522180	MF624251	n.a.	MF522179
*A. pararepens*	CCF 6352	MT193669	MT192308	n.a.	n.a.	n.a.
*A. pateriformis*	CGMCC3.27495 *	PP377805	PP373738	PP790575	PP790583	PP779724
*A. pateriformis*	SAUCC634702	PP377806	PP373739	PP790576	PP790584	PP779725
*A. pernambucoensis*	URM<BRA>7219	MN635568	MN635569	n.a.	n.a.	n.a.
*A. pseudocylindrospora*	EML-FSDY6-2	KU923817	KU923814	n.a.	KU923815	KU923819
*A. pseudocylindrospora*	CBS 100.62 *	NR_145276	MH869688	n.a.	n.a.	n.a.
*A. psychrophilia*	FSU4745	AY944874	EU736306	EU736252	AY944762	EU736279
*A. radiata*	CGMCC 3.16257	ON074698	ON074684	n.a.	n.a.	n.a.
*A. radiata*	XY09330-1	ON074699	ON074685	n.a.	n.a.	n.a.
*A. repens*	CBS 115583 *	NR_103624	NG_058551	n.a.	n.a.	n.a.
*A. saloaensis*	URM 8209 *	MN953781	MN953783	n.a.	n.a.	n.a.
*A. sichuanensis*	CGMCC 3.16258 *	NR_182589	ON074688	n.a.	n.a.	n.a.
*A. soli*	MFLU-20-0414 *	MT396373	MT393988	n.a.	n.a.	MT394049
*A. soli*	MFLU 20-0413	MT396371	MT393985	n.a.	n.a.	MT394046
*A. spinosa*	FSU551	AY944887	EU736307	EU736253	EU736227	EU736280
*A. stercoraria*	EML-DG8-1 *	KU168828	KT921998	KT922002	KT922000	NG_065640
*A. sympodialis*	CGMCC 3.16063 *	MZ354147	MZ350141	n.a.	n.a.	n.a.
*A. sympodialis*	CGMCC 3.16064	MZ354148	MZ350142	MZ357408	n.a.	n.a.
*A. terrestris*	FMR 14989 *	LT795003	LT795005	n.a.	n.a.	n.a.
*A. turgida*	CGMCC 3.16032 *	NR_189830	NG_241931	MZ357415	MZ357434	n.a.
*A. varians*	CGMCC 3.16065 *	MZ354149	MZ350143	MZ357409	MZ357428	n.a.
***A. *** ***verticilliformis***	CGMCC 3.27810 clone1 *	**PQ306315**	**PQ803170**	**PV126011**	**PQ807205**	**PQ799262**
***A. *** ***verticilliformis***	CGMCC 3.27810 clone2 *	**PV069755**	**PQ803171**	**PV126013**	**PV126025**	**PQ799263**
***A. *** ***verticilliformis***	**XG04088-4 clone1**	**PQ306316**	**PQ803172**	**PV126012**	**PQ807206**	**PQ799264**
***A. *** ***verticilliformis***	**XG04088-4 clone2**	**PV069756**	**PQ803173**	**PV126014**	**PV126026**	**PQ799265**
*A. virescens*	CGMCC 3.16066 *	MZ354150	MZ350144	MZ357410	MZ357429	n.a.
*A. virescens*	CGMCC 3.16067	MZ354151	MZ350145	MZ357411	MZ357430	n.a.
*A. xinjiangensis*	CGMCC 3.16107 *	OL678136	n.a.	n.a.	n.a.	n.a.
*A. yunnanensis*	XY09528	ON074701	ON074686	n.a.	n.a.	n.a.
*A. yunnanensis*	CGMCC 3.16259 *	NR_182591	NG_149054	n.a.	n.a.	n.a.
*A. zonata*	CGMCC 3.16033 *	NR_189831	MW671548	MZ357416	MZ357435	n.a.
*A. zygospora*	RSPG 214	KC478527	n.a.	n.a.	n.a.	n.a.
*A. zygospora*	ANG28	DQ914420	n.a.	n.a.	n.a.	n.a.
*Cunninghamella blakesleeana*	CBS 782.68	JN205869	MH870950	n.a.	n.a.	n.a.

Notes: New species proposed in this study are in bold. Ex-type or ex-holotype strains are labelled with an asterisk “*”. The abbreviation “n.a.” stands for “not available”.

## Data Availability

The sequences were deposited in the GenBank database.

## Supplementary Materials

The following supporting information can be downloaded at: https://www.mdpi.com/article/10.3390/microorganisms13061315/s1; File S1: ITS-SSU-LSU-*Act*-*TEF1a*; File S2: ITS.fas; File S3: Phylogenetic tree based on ITS; and File S4: ITS GenBank accession numbers.
