# Supplementary figures and images for: Unveiling Species Diversity Within Early-Diverging Fungi from China VI: Four Absidia sp. nov. (Mucorales) in Guizhou and Hainan
Source: Microorganisms. 2025 Jun 5;13(6):1315. doi: 10.3390/microorganisms13061315 (PMC12195128; doi:10.3390/microorganisms13061315)

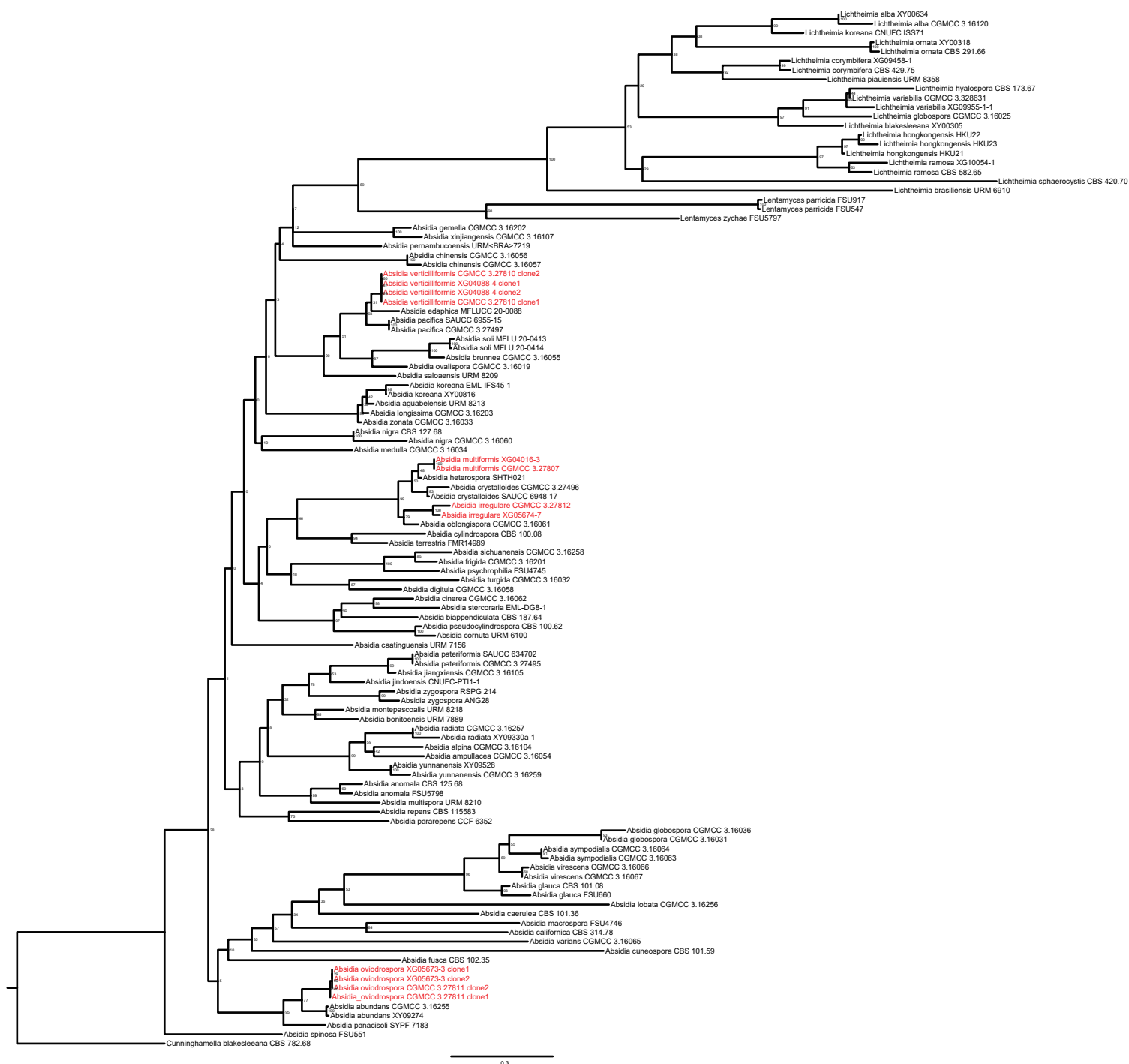

Supplement: Supplementary file 1 [file microorganisms-13-01315-s001.zip › S3 Phylogenetic tree based on ITS.pdf]
